# Modelling the linkage between influenza infection and cardiovascular events via thrombosis

**DOI:** 10.1038/s41598-020-70753-0

**Published:** 2020-08-31

**Authors:** Zachary McCarthy, Shixin Xu, Ashrafur Rahman, Nicola Luigi Bragazzi, Vicente F. Corrales-Medina, Jason Lee, Bruce T. Seet, Dion Neame, Edward Thommes, Jane Heffernan, Ayman Chit, Jianhong Wu

**Affiliations:** 1 Department of Mathematics and Statistics, York University, Toronto, ON Canada; 2 Laboratory for Industrial and Applied Mathematics, York University, Toronto, ON Canada; 3 Fields-CQAM Laboratory of Mathematics for Public Health, Fields Institute for Research in Mathematical Sciences, Toronto, ON Canada; 4 Centre for Disease Modelling, York University, Toronto, ON Canada; 5 Duke Kunshan University, Jiangsu, China; 6 Clinical Epidemiology Program, The Ottawa Hospital Research Institute, Ottawa, ON Canada; 7 Department of Medicine, University of Ottawa, Ottawa, ON Canada; 8 Sanofi Pasteur, Toronto, ON Canada; 9 Department of Molecular Genetics, University of Toronto, Toronto, Canada; 10 Sanofi Pasteur, Swiftwater, PA USA; 11 Department of Mathematics and Statistics, University of Guelph, Guelph, ON Canada; 12 Leslie Dan School of Pharmacy, University of Toronto, Toronto, ON Canada; 13 Department of Mathematics and Statistics, Oakland University, Rochester, MI USA

**Keywords:** Applied mathematics, Computational biology and bioinformatics, Infectious diseases

## Abstract

There is a heavy burden associated with influenza including all-cause hospitalization as well as severe cardiovascular and cardiorespiratory events. Influenza associated cardiac events have been linked to multiple biological pathways in a human host. To study the contribution of influenza virus infection to cardiovascular thrombotic events, we develop a dynamic model which incorporates some key elements of the host immune response, inflammatory response, and blood coagulation. We formulate these biological systems and integrate them into a cohesive modelling framework to show how blood clotting may be connected to influenza virus infection. With blood clot formation inside an artery resulting from influenza virus infection as the primary outcome of this integrated model, we demonstrate how blood clot severity may depend on circulating prothrombin levels. We also utilize our model to leverage clinical data to inform the threshold level of the inflammatory cytokine TNFα which initiates tissue factor induction and subsequent blood clotting. Our model provides a tool to explore how individual biological components contribute to blood clotting events in the presence of influenza infection, to identify individuals at risk of clotting based on their circulating prothrombin levels, and to guide the development of future vaccines to optimally interact with the immune system.

## Introduction

Influenza infection is associated with severe cardiovascular and cardiorespiratory events^1–5^. An increased risk of acute myocardial infarction has been observed within 7 days of influenza infection^1^. Several in vivo experiments in humans and animals found extensive and profound procoagulant effects due to influenza infection^6^. A positive association between influenza infection and stroke has also been observed^7^. Biological mechanisms explaining the various cardiovascular events associated with influenza have recently been proposed; however, a quantitative framework capturing these biological mechanisms to acute myocardial infarction (AMI) and stroke has not yet been established^7^. A model that can assist in predicting the risk of AMI and stroke arising from influenza infection is important to inform prevention and treatment strategies (such as vaccination) and enhance risk-assessment capabilities. This technical challenge offers an opportunity for mathematical modelling to play a role in filling this key knowledge gap.

Mathematical models describing the viral dynamics of influenza infection incorporate a variety of biological detail and data utilization, incorporating either in vitro or in vivo data sets (for a comprehensive recent review, see^8^). Similarly, extensive research has gone into the modelling, experimentation, and study of the dynamics of blood coagulation^9–15^ (for a review, see^16^). Despite the connectedness of the post-infection viral dynamics, immune response, and blood coagulation, no modeling study has been conducted to link these systems together. Here, we develop a comprehensive mathematical model capturing the major components of the probable biological mechanisms, with an objective of linking influenza infection to thrombotic cardiovascular events. This framework incorporates the modelling and synthesis of three key events; immune response to influenza infection, subsequent inflammation, and blood coagulation. We have identified tissue factor (TF) as the critical component linking inflammation to coagulation^17,18^. Hence, we model the induction of tissue factor arising from influenza virus infection. Our model incorporates this critical factor, TF, to establish a quantitative link between influenza infection and its effects triggering the blood coagulation cascade. As coagulation also plays a key role in arterial thrombosis, our integrated framework develops a model and analysis establishing linkage between influenza infection and arterial thrombosis^19^.

In light of the observed relationships between influenza infection and outcomes such as stroke and AMI^1^, we focus in this study on blood clots forming within arteries (arterial thrombosis). Although there are several pathways by which influenza may induce cardiovascular events, we focus on blood clot severity as a proxy for AMI or stroke risk^19^. Hence the outcome of our model is the size of arterial blockage which may form following influenza infection. Through the modelling of the crucial biological components arising from acute infection, such as IFN-I, CD8^+^ T Cells, TNF$\alpha$, thrombin, and inflammatory cytokines^8^, we obtain several key qualitative and quantitative results. We estimate the threshold level of tissue factor (III) for which, if surpassed, a culprit atherosclerotic plaque will be disrupted and formation of a blood clot within an artery will occur. Furthermore, our model predicts that the resulting blood clot size following influenza infection is positively associated with circulating prothrombin ([II]) levels, which are affected by various genetic conditions and vary person-to-person^20^.

The rest of the paper is organized as follows: we present the key study results including an overview of the mathematical model structure, model simulations illustrating the key events leading to a blood clot, as well as the effects of circulating prothrombin levels on blood clot size. We also demonstrate how our model may provide an estimate for the threshold amount of inflammation (for which we use TNF$\alpha$ as a proxy) required to initiate blood coagulation. Zooming out, we discuss several applications of our work as well as underlying assumptions and limitations of the mathematical model developed herein. In addition, we outline a number of opportune directions for future research.

## Results

### Formulation of model framework to model a linkage between influenza infection and blood clotting

**Figure 1:**
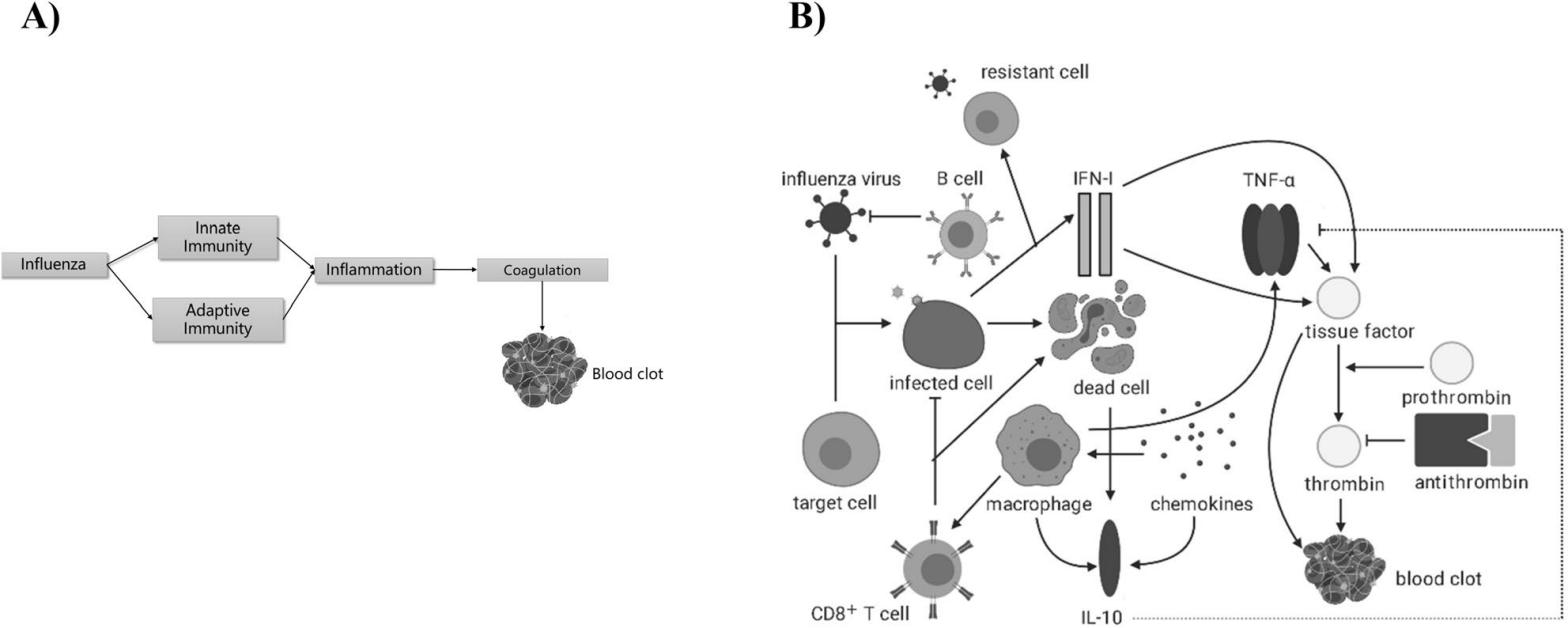
Diagrams of Model (1): (**A**) Key events and systems involved in the possible disruption of a culprit atherosclerotic plaque and subsequent blood clot formation following influenza infection. Figure created using Microsoft PowerPoint, Version 2006 (https://www.microsoft.com/en-ca/microsoft-365/powerpoint). (**B**) Schematic diagram illustrating the model variables and primary relationships between them. Solid lines represent direct production or stimulation as assumed in model (1). Dotted lines represent enhanced production or synergism (i.e., the enhanced production of TNF $\alpha$ in the presence of IL-10). See Methods for details of model development and its formulation. Figure created using BioRender (https://biorender.com/).

The backbone of our study is a mathematical model, which we leverage to study blood clotting within an artery as a consequence of influenza infection. We modelled the infection process using a system of ordinary differential equations (ODEs). In the model, we capture the viral infection process consisting of the dynamics of viral load, uninfected target epithelial cells, and infected target epithelial cells. Our model also describes the immune and inflammatory responses to infection. In particular, our model includes type I interferon (IFN-I), comprising a key portion of the innate immune response and CD8^+^ T cells, a key component of the adaptive immune system. We link inflammation to blood coagulation and subsequent clotting through the modelling of TF, a distinguishing feature of this study. Within this modelling framework, blood coagulation initiates the disruption of a culprit atherosclerotic plaque. The biological systems involved in key events leading to clotting are depicted in Fig. 1A. A schematic of the model structure illustrating the key model components and their relationships is shown in Fig. 1B.

### Model simulation results

#### Model consistency and time dynamics

Overall, the viral dynamics and resulting immune responses against influenza inflammation generated from our model are in agreement with existing models of similar scope^21,22^. The integrated model dynamics corresponding to a single influenza infection and the possible subsequent clotting events are displayed in Supplementary Figure S1 and S2 in the Supplementary Information (SI).

#### Estimation of threshold amount of TNF$\alpha$ to induce tissue factor

**Figure 2:**
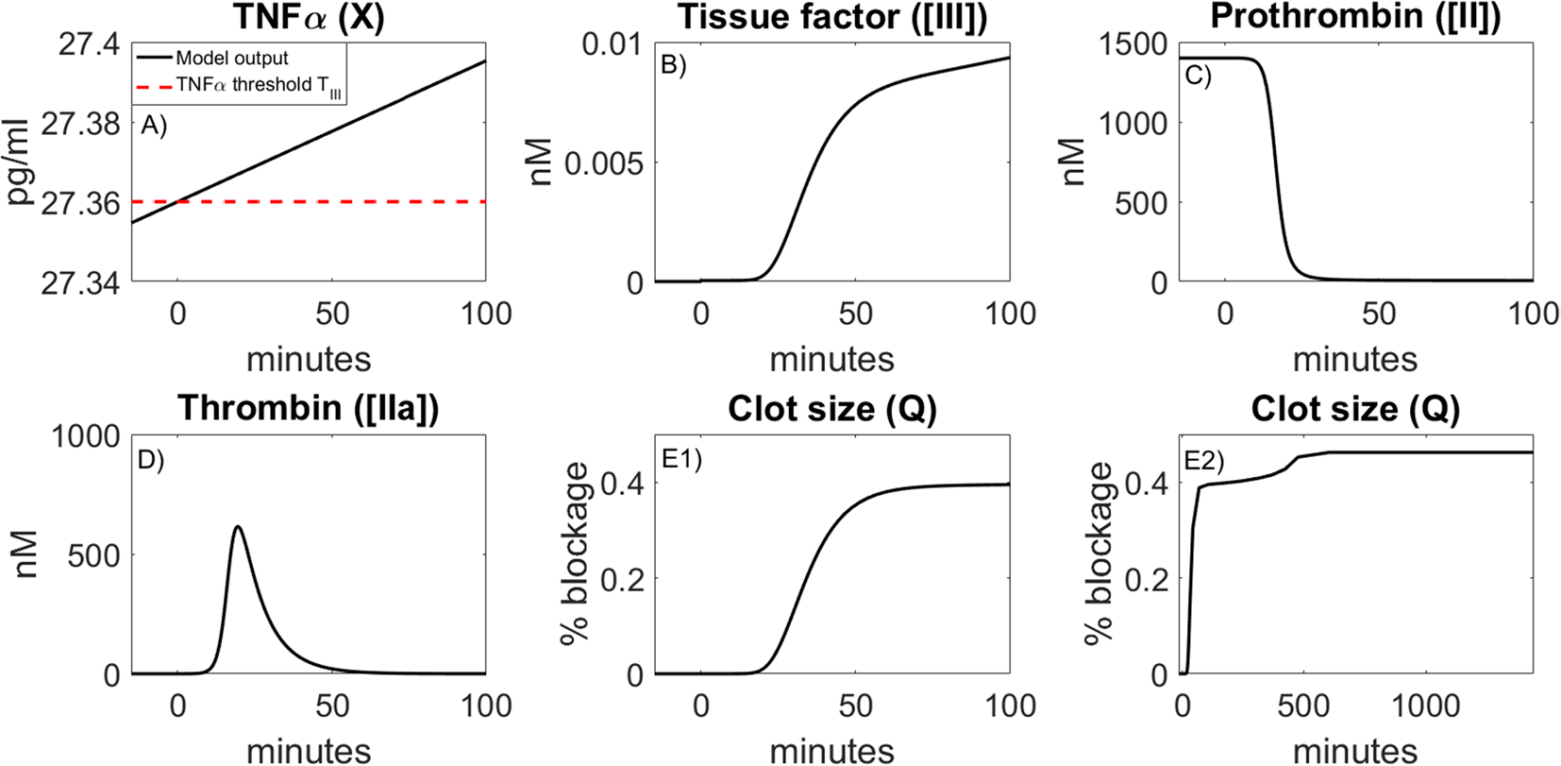
Figure illustrates key events leading to a blood clot: (**A**) TNF $\alpha$ induced from influenza infection rises above the threshold level ${T}_{III}$, resulting in the induction of tissue factor (TF) expression at the site of a vulnerable atherosclerotic plaque (**B**). With TF present (**C**, **D**), the blood coagulation cascade begins and, simultaneously, prothrombin is consumed while thrombin is generated. TF exposure and thrombin presence allow for the formation of a blood clot (**E1** and **E2**). This blood clotting event results in approximately 0.45% arterial blockage. (**E2**) The clot’s percentage obstruction over the course of 1,440 min (24 h) period. The two distinct growth phases of the blood clot are made apparent over this 24-h span (see “Estimation of threshold amount of TNFα to induce tissue factor” section for interpretation).

Using our mathematical model, we leverage recent clinical observations^1^ to estimate the threshold amount of the inflammatory cytokine TNFα required to initiate TF induction and subsequent clotting at the site of a vulnerable atherosclerotic plaque. In particular, we estimate this concentration of TNFα to be T_III_ = 27.36 pg/ml. This threshold T_III_ may provide a benchmark for determining a patient’s risk of cardiovascular event, which we elaborate on the potential clinical applications of T_III_ in the discussion. We also highlight its key role in Fig. 2; once TF is induced, the blood coagulation cascade occurs, and blood clot formation begins. For the details and strategy for estimating T_III_ see Supplementary Information (SI), section S1.2.

We also note that atherosclerotic plaque disruption and blood clotting may not necessarily occur following influenza infection. We capture this key fact with mathematical model (1); not all trajectories following infection result in blood clot formation. Many of the model parameters (representing rates of underlying biological mechanisms, cell counts, concentrations, and other biological properties) are implemented as ranges, rather than single values. Ranges are used to reflect estimated uncertainty in their true values. Therefore, each model run will produce different results (or trajectories).

Next, we illustrate the clotting event and the connecting links from inflammation to coagulation. When TNFα levels rise above the threshold T_III_, TF expression is induced and the blood coagulation cascade begins, resulting in the disruption of a culprit atherosclerotic plaque and subsequent blood clot formation. We highlight this defining feature of model (1) in Fig. 2 and note that blood clots are formed rapidly following thrombin generation. The resulting clotting time is about 75 min (Fig. 2 (E1)), in agreement with prior in vitro experimental works^10^*.* We also depict blood clot size over the course of 1,440 min (24 h) to illustrate the long-term clotting behavior (Fig. 2 (E2)). Following initial blood clot formation there is a local accumulation of procoagulants such as TNFα and IL-17, platelets, and these factors contribute to the continued growth of the blood clot until the growth phase ends^23^*.* The model trajectories reflect this biphasic behavior as following blood clot formation, procoagulants TNFα and TF levels increase in time. The growth phase end is a result of thrombin depletion.

#### Effect of circulating prothrombin levels on the artery blockage

**Figure 3:**
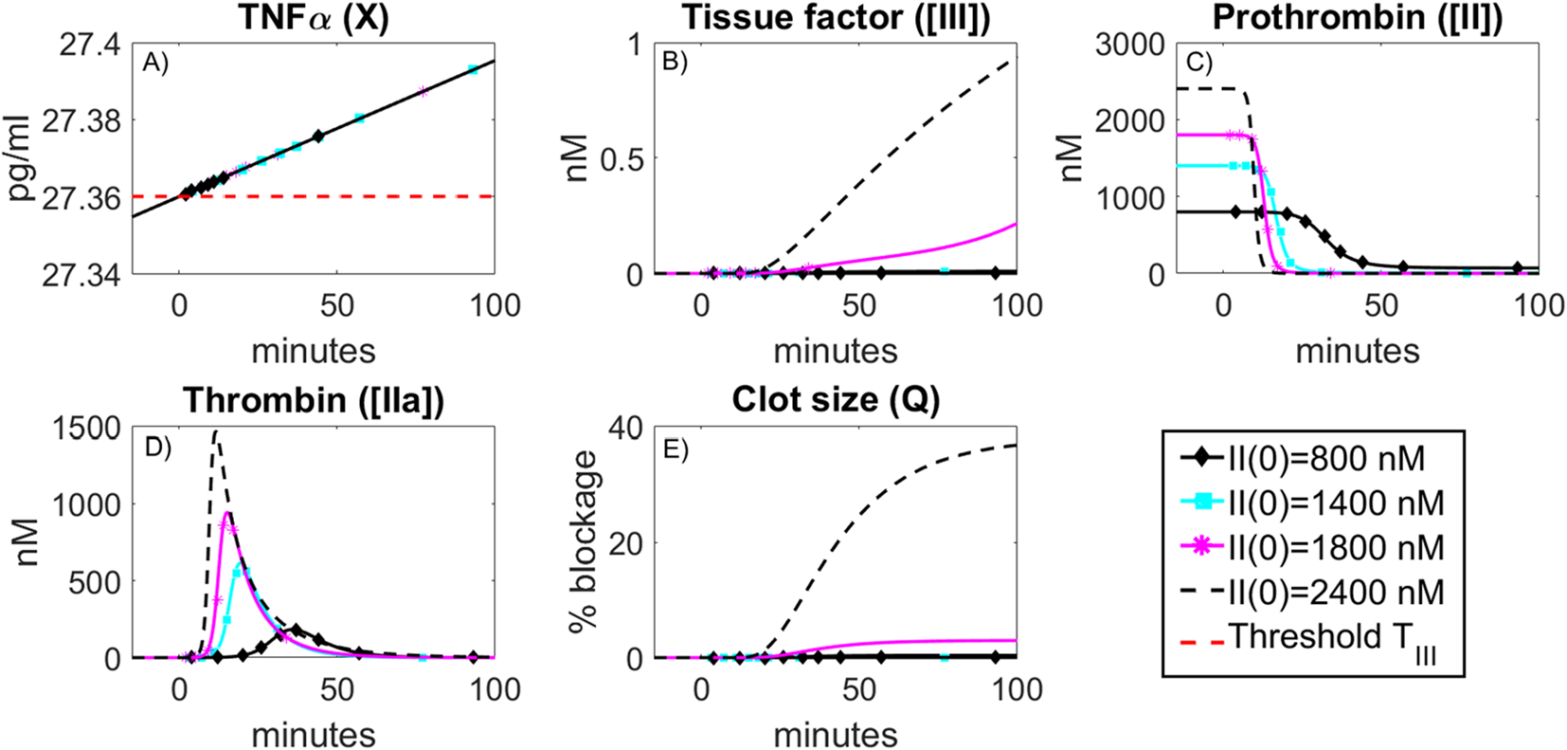
Effect of circulating prothrombin levels on clot size: For a brief description of the events leading to a blood clot in Panels (**A**–**E**), see Fig. 2. Panels (**C**, **D**) Initial values of prothrombin influence the size of the thrombin peak. This results in blood clot size dependence on the initial prothrombin level. Prothrombin levels in blood vary person-to-person and the risk of blood clot formation may depend accordingly^20^. Model (1) may provide the ability to quantify these differences in severity and subsequent risk. Panel (**E**) Resulting blood clot sizes due to varying circulating prothrombin levels. As initial prothrombin level increases, resultant blood clot size increases. As a result, model (1) may be used to quantify the role of prothrombin in determining blood clot severity.

One prediction of model (1) is the positive association between blood clot severity and circulating prothrombin levels (illustrated in Fig. 3). High prothrombin levels have been correlated with a risk of arterial thrombosis and are also affected by genetic conditions, hence vary person-to-person^20^*.* We show how the blood clot severity, as predicted by model (1), depends on circulating prothrombin levels. However, the model-predicted likelihood of blood clotting formation following influenza infection remains constant. An influenza infection may not induce blood clot formation as TNF$\alpha$ can remain below the threshold ${T}_{III}$ post-infection. In other words, the individual’s inflammatory state arising from infection may not be sufficient to disrupt an existing atherosclerotic plaque. We also note that a positive association between the magnitude of the thrombin peak and prothrombin levels have been observed^20^ and model (1) is in agreement, also capturing this association.

## Discussion

In this work, we present the development and simulation of a mathematical model that connects influenza virus infection to subsequent blood coagulation, blood clotting risk, and blood clotting severity. Our aim is to lay a foundation by modelling the risk of cardiovascular thrombotic events resulting from acute influenza infection, incorporating mechanistic details of the process. This foundational framework features modularity allowing for augmentation, expansion and future study.

Our primary contributions in terms of modelling are the connections linking the inflammatory response and blood coagulation. The model dynamics presented agree with several existing studies and the model produces biologically tractable viral trajectories^21,22^*.* Further, the blood clotting behavior generated from our integrated model captures many qualitative and quantitative features of in vitro experimentation by incorporating mechanistic biological detail^10^. Procoagulant effects observed in vivo in terms of their association with influenza infection are consistent with the model simulations^6^. This modelling framework is amenable to risk assessment and may be used to quantify the effects of influenza vaccination on subsequent blood clotting event risk and severity. This mathematical model may also be used to identify key parameters which contribute (positively or negatively) to blood clotting events (e.g. with sensitivity analysis). In other words, we may use the model to infer which particular biological actions/feedbacks are most crucial to blood clotting. One consequence of the model developed herein is that regulating TNFα also impacts the likelihood of developing a blood clot. As a result, we postulate that vaccine-induced TNFα regulation may reduce the chance of blood clot formation. Specifically, vaccines that aim to induce an immune response resulting in the production of antibodies that neutralize inflammatory cytokines (e.g. TNFα, IL-1β) may be weighed in terms of their impact on risk of cardiovascular events^24–26^. Currently, a phase I clinical trial of a vaccine targeting IL-1β has been completed as well as a phase IIa clinical trial for an anti-TNF vaccine^25,26^. In this light, our model may be used to quantify the effects of anti-cytokine therapy (e.g. anti-TNF therapeutics for rheumatoid arthritis) on the risk of cardiovascular events. Anti-inflammatory drugs exist; however, have several drawbacks making vaccines a potential attractive option in the future^27^*.* In addition, vaccines or adjuvants aiming to induce a T-cell response may be weighed in terms of the resulting cardiovascular event risk. Overall, this framework may prove to be useful for guiding ideal vaccine design and conducting scenario analyses. We also note that the model and parameter estimates may be implemented and made available in a user-friendly format (e.g. software package, web-based tool, etc.).

This model quantifies the size of a blood clot resulting from influenza infection. We restrict our study scope to arterial thrombosis arising from acute infection, as we are interested in quantifying AMI and stroke risk. We do note that our modelling framework is also amenable to the study of venous thrombosis. Whether the size of the blood clot translates to risk of thrombotic cardiovascular events (e.g. AMIs) is not clear at the present time. Similarly, the extent of these effects of blood clot size on thrombotic cardiovascular event risk has not yet been established to our best knowledge. Currently, the relationship between blood coagulation and blood clotting is informed using experimental in vitro data measuring clot size within a flow chamber and cannot exhibit cardiovascular events. On the other hand, humans can exhibit observable cardiovascular events; however, to our best knowledge we do not have the ability to dynamically and consistently measure clot size within a blood vessel or artery in a living patient. For these reasons, a quantitative link between blood clot size and thrombotic cardiovascular risk may not be tractable at the present time, although it is an important subject for future studies.

A key result of our work is the estimation of the threshold TNFα level required to initiate TF expression, T_III_. We estimate T_III_ to be 27.36 pg/ml, which lies above the average clinical measurements of normal adult subjects with no significant illnesses (TNFα = 0.7 ± 0.2 pg/ml, n = 28 subjects)^28^*.* This threshold, T_III,_ also may distinguish TNFα levels between control adults with mild or moderate infection (TNFα = 14.80 ± 4.74 pg/ml) and patients with chronic liver failure (TNFα = 53 ± 74.4 pg/ml in surviving patients and TNFα = 87.76 ± 91.38 pg/ml among non-survivors)^29^. These clinical observations serve as a form of validation, as T_III_ may lie between TNFα levels of healthy individuals and those at-risk individuals with more severe complications (e.g., chronic liver failure). This threshold may be used in a clinical setting using patient's TNFα measurements taken over time as a proxy for risk. An observed increase or decrease in TNFα measurements may indicate cardiovascular distress and systemic inflammation, hence a change in risk of blood clotting may be inferred from this systemic measurement. In this sense, TNFα and threshold T_III_ may be used as a biomarker for cardiovascular risk. We also note that this threshold may depend on IFN-I levels, IL-1β, etc.

There are several simplifications made within this study to develop and illustrate our framework. The extrinsic blood coagulation system involves many additional interactions and components, which should be considered in future studies. Realistically blood clotting depends on spatial heterogeneity in blood plasma, blood flow velocity, fibrin, Factor IXa, Factor Xa, etc.^20,28^. We conducted our study for the case where atherosclerotic plaques are static elements, in fact they are complex and dynamic structures whose cellular and extracellular make-up may significantly affect the risk of cardiovascular atherosclerotic events^30^. In our study, TF is induced primarily by TNFα and IFN-I, with synergism from thrombin. TF induction is inhibited and regulated by many inflammatory cytokines, chemokines, and enzymes that should be considered in future studies^31,32^*.* In this study, we focus on cardiovascular outcomes immediately following acute influenza infection. As a result, we make a simplifying assumption that prothrombin is not replenished naturally and therefore, a host may exhibit a maximum of one clotting event. In the current work, we assumed that when sufficient concentration of TNF$\alpha$ is present, which we take as proxy for inflammation, then TF is induced resulting in the disruption of an existing atherosclerotic plaque and subsequent blood clotting occurs. Lastly, we assume that a minimum amount of TNFα is required to induce TF. Once this amount of TNFα exceeds this threshold, TF is exposed. We expect the magnitude of such a threshold as T_III_ to vary based on the health status of individuals which may be verified clinically. We expect that this threshold is higher for young individuals compared to older individuals or those with underlying health conditions, who may be more susceptible to blood clotting. The alternative case is also possible; older adults and those with underlying health conditions may have a higher threshold. At-risk individuals likely have high resting TNFα levels, perhaps even higher than the 27.36 pg/ml threshold. Although this mechanistic study has neglected many components and interactions involved in the complex immune response, inflammatory response, and blood coagulation systems, we believe that we have established a flexible framework amenable to further augmentation and modification to account for further biological realism.

### Future directions

The existing model (1) may be augmented to incorporate specific biomarkers for inflammation (e.g. IL-6), matrix metalloproteinases (MMPs), neutrophil activity, and platelet activity. The sensitivity of the primary biological actions and feedbacks impacting these markers on systemic inflammation and clot risk may be explored. Clinical measurements of these markers may also be used to assist with further study. Along these lines, we note that many model variables are proteins and cells which are routinely measured and there is a large amount of quantitative data for. In future studies, we shall use these cell and protein counts for model calibration, testing, and validation. We will add more physiological realism to the blood coagulation and blood clotting submodels, including feedbacks within the blood clot formation and inflammatory system^13,33^. Specifically, interleukins and TNFα may be induced as a result of blood clot formation. Also, this model and framework may be coupled to spatially heterogeneous coagulation and clotting models (e.g. ^23,30,34–36^). Future work may aim to estimate ${T}_{III}$ based on a groups of varying health status, such as high-risk groups. The impacts of pre-existing TNF$\alpha$ on the timing and likelihood of blood clot formation may also be explored in follow-up works. Lastly, current influenza vaccines mainly aim at generating antibody responses to the virus, but a vaccine that yields flu-protective T cells, which would provide longer lasting memory responses, is lacking^37,38^. Therefore, this study may provide new insights that inform new strategies to develop more efficient vaccines that drive flu-protective T cell responses and novel antiviral therapies for influenza infections.

## Methods

### Model development

We develop the mathematical model linking viral infection to the immune and inflammatory response, blood coagulation, and subsequent blood clot formation using a system of ordinary differential equations. Based on biological evidence and prior modelling studies, we formulate a set of modelling assumptions to develop Model (1)^10,12,15,18,21–23,27,31–34,39–47^. The model is given below without detailed interpretation and development. The term-by-term mathematical formulation and detailed explanations of model assumptions are given in Section S1.1 in the Supplementary Information (SI). A list of the model variables, and units of measurement are also given in Supplementary Table S2. In this work, a model variable refers to the components of the biological system dynamically evolving over time. The model parameters broadly represent rates which characterize the evolution of the system.

Model (1): A mathematical model describing the influenza infection process and resulting immune response, inflammatory response, blood coagulation, and subsequent blood clotting:

1 $$V^{\prime} = p_{v} I - \delta_{V} V - \beta VT - g_{va} VB$$

2 $$T^{\prime} = g_{t} \left( {T + R} \right)\left( {1 - \frac{T + R + I}{{T_{0} }}} \right) - \beta^{\prime}VT + \rho R - \phi FT$$

3 $$I^{\prime} = \beta^{\prime}VT - \delta_{I} I - k_{N} IF - k_{E} CI$$

4 $$F^{\prime} = p_{F} I - \delta_{F} F$$

5 $$R^{\prime} = \phi FT - \rho R$$

6 $$C^{\prime} = \frac{{b_{cp} M^{{h_{c} }} }}{{a_{cp}^{{h_{c} }} + M^{{h_{c} }} }} - b_{ei} R_{F} IC - \mu_{C} C$$

7 $$B^{\prime} = b_{P} + b_{BP} ML\left( {b_{max} - B} \right) - \mu_{B} B$$

8 $$L^{\prime} = \frac{{b_{L} M\Sigma_{1} }}{{\Sigma_{1} + \left( {\frac{{g_{1} L + g_{2} }}{{L + d_{2} }}} \right)}} - \mu_{L} \left( {L - b_{LT} \left( {1 - R_{F} } \right)T} \right)$$

9 $$D^{\prime} = \delta_{I} I + k_{N} IF + k_{E} CI$$

10 $$X^{\prime} = \frac{{b_{x} M\Sigma_{2} }}{{\Sigma_{2} + \left( {\Sigma_{2} + \frac{{g_{1} L + g_{2} }}{{L + d_{2} }}} \right)\left( {\frac{{k_{1} L + k_{2} }}{{L + d_{1} }}} \right)}} - \mu_{X} X$$

11 $$M^{\prime} = \frac{{b_{mk} K^{{h_{k} }} }}{{a_{mk}^{{h_{k} }} + K^{{h_{k} }} }} - \mu_{M} \left( {M - b_{M} } \right)$$

12 $$K^{\prime} = \frac{{b_{K} M\Sigma_{1} }}{{\Sigma_{1} + \frac{{\left( {g_{1} L + g_{2} } \right)}}{{L + d_{2} }}}} - \mu_{k} K$$

13 $$\left[ {III} \right]^{\prime} = \frac{{\lambda_{III} \left( {X + F} \right)}}{{h_{III} + \left( {\frac{{g_{4} }}{{\left[ {IIa} \right] + d_{IIa} }}} \right) + X + F}} - K_{S} \left[ {II} \right]\left[ {III} \right] - \mu_{III} \left[ {III} \right]$$

14 $$\left[ {II} \right]^{\prime} = - K_{S} \left[ {II} \right]\left[ {III} \right] - K_{P} \left[ {II} \right]\left[ {IIa} \right]$$

15 $$\left[ {IIa} \right]^{\prime} = K_{S} \left[ {II} \right]\left[ {III} \right] + K_{P} \left[ {II} \right]\left[ {IIa} \right] - K_{I} \left[ {IIa} \right]\left[ {AT} \right]$$

16 $$\left[ {AT} \right]^{\prime} = - K_{I} \left[ {IIa} \right]\left[ {III} \right]\left[ {AT} \right]$$

17 $$Q^{\prime} = \kappa \left[ {IIa} \right]\left[ {III} \right]\left( {1 - Q} \right)$$

where $$\Sigma_{1} = a_{11} X + a_{12} D$$, $$\Sigma_{2} = a_{11} X + a_{12} D + \frac{{a_{21} V}}{{a_{22} + V}}$$, and $$R_{F} = \frac{F}{{a_{RF} + F}}$$.

$${\text{TF induction threshold T}}_{{{\text{III}}}} :{ } \lambda_{III} = \left\{ {\begin{array}{*{20}c} {0, \hspace{1.35cm} X < T_{III} } \\ {\lambda_{III0} , \hspace{0.5cm} X \ge T_{III} } \\ \end{array} } \right.$$

**Model variables: **V = Virus (EID_50_/ml); T = Target epithelial cells (Cells); I = Infected epithelial cells (Cells); F = Type I interferon (dimensionless (We invite the reader to Supplementary Information (SI) S1.3 explaining the dimensions of IFN-I.)); R = Resistant target epithelial cells (Cells); C = CD8^+^ T cell (Cells); B = B cell (Cells); L = Interleukin-10 (pg/ml); D = Dead cell (Cells); X = TNF $\alpha$ (pg/ml) ; M = Macrophage (Cells); K = Chemokines (Cells); [III] = Tissue factor (nM); [IIa] = Thrombin (nM); [II] = Prothrombin (nM); [AT] = Antithrombin (nM); Q = Blood clot size (% arterial blockage).

### Data and model parameter estimation

We inform parameters for model (1) using data, existing models, and experimental findings from several sources^1,12,21–23,27,46,48^. We use model parameter values from existing influenza viral dynamics modeling studies^21,22^, blood coagulation modeling studies^12,23^. We determine remaining parameters by integrating several additional sources of experimental and clinical data. For the details of parameter estimation see the parameter estimation process in the Supplementary Information (SI) (section S1.2). The model parameters which inform the mathematical model (1) along with their respective ranges, units, and sources are in the Supplementary Information (SI) Supplementary Table S3.

### Model simulation

To generate Figs. 2 and 3, Supplementary Figures S1 and S2, we first inform model (1) with parameters from their baseline values in Supplementary Table S3 and initial conditions in Supplementary Table S2. We then solve the corresponding system of ordinary differential equations numerically using Matlab’s ode45, an implementation of the Runge–Kutta method. We consider an uninfected individual at baseline and simulate the infection process numerically. In other words, we consider the system state initially at the disease free equilibrium of model (1) with an initial viral load, reflected in initial conditions shown in Supplementary Table S2 in the Supplementary Information (SI).

## Supplementary information

Supplementary file1

## Data Availability

No datasets were generated or analyzed during the current study.
