# Supplementary material for: Modelling the linkage between influenza infection and cardiovascular events via thrombosis
Source: Sci Rep. 2020 Aug 31;10:14264. doi: 10.1038/s41598-020-70753-0 (PMC7458909; doi:10.1038/s41598-020-70753-0)
Supplement: Supplementary file 1 — Supplementary file1 [file 41598_2020_70753_MOESM1_ESM.pdf]

Modelling the linkage between influenza infection and cardiovascular events via thrombosis

Authors: Zachary J. McCarthy, Shixin Xu, Ashrafur Rahman, Nicola Luigi Bragazzi, Vicente F. Corrales-Medina, Jason Lee, Bruce T. Seet, Dion Neame, Edward Thommes, Jane Heffernan, Ayman Chit, Jianhong Wu

S1. Supplementary Methods

S1.1 Model development

We model the influenza infection process using a system of ordinary differential equations (ODEs). Model variables and units are given in Figure 1 Legend. Similarly, parameter values and corresponding descriptions are provided in Supplementary Table S3. A schematic of the model structure illustrating the key model components and their relationships are shown in Figure 1A.

Equations (1)-(3) describe the basic infection process consisting of the viral load, uninfected target cells, and infected target cells. Equations (4)-(12) describe the immune and inflammatory response to infection. In particular, Equation (4) represents IFN-I comprising the key portion of the adaptive immune response and Equation (6) representing $CD8^+$ T cells which is a key component of the adaptive immune system. Equations (1) through (12) are adapted from existing models [21], [22].

S1.1.1 Model for influenza infection, immune response, and inflammatory response.

We model the change in virus V with Equation (1). The first term $(p_v I)$ represents the increase of virus from infected cells, $(\delta_V V)$ represents the natural death of virions, and (βVT) represents depletion of virions due to infecting target cells. Lastly, the term $(g_{va} VB)$ represents the inactivation of virions due to antibodies.

We describe target epithelial cells T with Equation (2). The first term in this equation represents logistic growth with total target cell population $T + R + I$ limited by carrying capacity T_0. The second term $(\beta' VT)$ represents infection of target cells due to virus. Resistant target cells lose protection at rate ρ, hence becoming susceptible to infection. Lastly, target cells become resistant in the presence of IFN-I represented by the last term (ϕFT). Equation (3) represents the change in infected target cell population I. Virus infects target cells hence the term $(\beta' VT)$ reflects the resulting increase in infected cells. Infected cells decay naturally with death rate δ_I. The term $(k_N IF)$ represents IFN-activated natural killer (NK) cells neutralizing infected cells [22]. This is important because NK cells limit the early dissemination of the virus by killing infected cells and secreting inflammatory and antiviral cytokines like IFN−I to activate macrophages. Lastly, $(k_E CI)$ models the decay of infected cells by $CD8^+$T cells, which come from the adaptive immune response [22]. $CD8^+$ T cells play a crucial role in anti-viral immunity, but current influenza vaccines do not lead to a robust and long-lasting $CD8^+$ T cell memory response.

Therefore, focusing our model on these cells provides critical insights into how future vaccines should be developed to improve flu-specific T cell responses. Equation (4) models the innate immune response given by IFN-I (F). Here infected cells promote the innate immune response and IFN-I decays at rate δ_F. Equation (5) represents the resistant target cell population and follows directly from Equation (2). $CD8^+$ T cells (Equation 6) are activated by macrophages, whose growth we model with a Hill function (with Hill coefficient h_c). $CD8^+$ T cells are depleted by inactivating infected target cells, reflected in the second term $(b_{ei}R_F IE)$, and decay at rate μ_C [21]. Equation (7) models the B cell population. B cells exist naturally, which we capture with the recruitment b_p and decay μ_B rates. B cell production, reflected in the term $b_{BP}ML(b_{max} - B)$ is also stimulated by macrophages and IL-10 [21]. Equation (8) models the change in IL-10. The first term captures the production of IL-10 from macrophages. IL-10 (L) and Chemokines (K) are produced in a similar way by macrophages. Due to the receptor saturation in macrophages the Michaelis-Menten saturation term is used to model their growths. IL-10 inhibits its growth by itself in a saturation fashion. The second term $\mu_L(L - b_{LT}(1 - R_F)T)$ of (8) represents the decay of IL-10 at rate μ_L and production from target cells in the lung, which is limited by presence of IFN-I [21]. Equation (9) models the change in the dead cells, which follows from equation (3). Note that we only include dead and do not consider damaged epithelial cells in this study. Equation (10) models the change in TNFα, denoted by X, whose production is stimulated by the interaction of macrophages with the presence of viral particles, exogenous TNFα, and dead cells. For the details of the TNFα production see [21]. Equation (11) models macrophages (M), which are comprised of *i)* macrophages which persist in epithelial tissue (i.e. tissue resident macrophages) and *ii)* macrophages that enter the lung from blood via a chemokine gradient (i.e. inflammatory macrophages) [21]. Tissue resident macrophages are essential for initiating the inflammatory response and recruitment of inflammatory cells to the infection site, including NK cells and monocytes, which ultimately differentiate into inflammatory macrophages. Equation (12) models the production of chemokines (K). Macrophages and pro-inflammatory signal Σ_1 stimulate chemokine production. We also consider the effects of IL-10 and its inhibition of chemokine production [21]. IL-10 mediated regulation of the inflammatory process is important for limiting tissue damage associated with inflammation and initiating tissue repair.

Finally, we note explicitly that TNFα production is stimulated by the accumulation of pro-inflammatory signal Σ_2, whose strength increases with the accumulation of dead cells, influenza virus, and TNFα itself. Similarly, IL-10 and chemokines are stimulated by the pro-inflammatory signal Σ_1. The strength of the signal Σ_1 increases with the accumulation of dead cells and influenza virus.

S1.1.2 Model for blood coagulation

Linking inflammation to blood coagulation via TF induction is a key feature of this study. In Figure 1A and Figure 1B we depict the key events considered in this study leading to a blood clot. Inflammatory cytokines, such as tumor necrosis factor TNFα, lipopolysaccharides (LPS), IL-1, c-reactive protein, and IFN-Iβ induce the production of tissue factor [21]. Thrombin, which is generated during blood coagulation, has a synergistic effect on TF production [10,12, 18, 40, 41, 49 - 51]. On the other hand, anti-inflammatory cytokines such as IL-10 regulate the activity

of tissue factor [31]. TF will trigger the blood coagulation process and prothrombin will be consumed. Thrombin is produced as a result of the activation and consumption of prothrombin [14, 34]. Meanwhile, thrombin could also enhance the activation of thrombin when a sufficient concentration is present [14, 34].

We connect the inflammatory response and blood coagulation via TF induction. Here we model TF production by Equation (13). The first term on the right-hand side of Equation (13) accounts for the synergistic effects of thrombin-TNFα and induction from IFN-I. The second term ($K_S[II][III]$) takes into consideration the activation of prothrombin ([II]) and the last term is natural degradation at rate μ_{III}. Here the growth rate λ_{III} is set to 0 if the TNFα concentration is below a threshold T_{III}. When TNFα passes this threshold concentration level, an existing atherosclerotic plaque will be disrupted, resulting in the exposure of TF and formation of a blood clot [52]. With TF induced, blood coagulation begins and thrombin generation occurs. Equations (14)-(16) model the prothrombin, thrombin, and antithrombin dynamics, respectively. We adopt a model of the simplified extrinsic pathway describing thrombin generation or blood coagulation cascade [12]. In particular, we adopt a model of the simplified extrinsic pathway due to the size and complexity of the mechanistic physiological models (e.g. [9, 11]). The initiation phase consists of consuming existing prothrombin which forms thrombin at rate K_S. In the propagation phase, thrombin generation from prothrombin occurs at rate K_P. Finally, thrombin inhibition is modelled using the rate constant K_I [12]. The parameters K_S, K_P, K_I are switched on and off based on the amount of thrombin present and as a result are piecewise function (see Supplementary Table S1 for the switching conditions) [12].

Finally, we model the size of a blood clot resulting from active thrombin with Equation (17). We assume, in addition to thrombin presence, the growth of a blood clot requires the presence of TF ([III]). Here Q represents the percentage of an artery that is restricted by a blood clot. To capture qualitative behavior of blood clot formation, we ensure that Q is bounded above by 1 and also increases in the presence of thrombin and prothrombin [9].

Model (1): A mathematical model describing influenza infection process and resulting immune response, inflammatory response, blood coagulation, and subsequent blood clotting:

$$V' = p_v I - \delta_V V - \beta VT - g_{va} VB \qquad (1)$$

$$T' = g_t (T + R)(1 - \frac{T+R+I}{T_0}) - \beta' VT + \rho R - \phi FT \qquad (2)$$

$$I' = \beta' VT - \delta_I I - k_N IF - k_E CI \qquad (3)$$

$$F' = p_F I - \delta_F F \qquad (4)$$

$$R' = \phi FT - \rho R \qquad (5)$$

$$C' = \frac{b_{cp} M^{h_c}}{a_{cp}^{h_c} + M^{h_c}} - b_{ei} R_F IC - \mu_C C \qquad (6)$$

$$B' = b_P + b_{BP}ML(b_{max} - B) - \mu_B B \qquad (7)$$

$$L' = \frac{b_L M \Sigma_1}{\Sigma_1 + (\frac{g_1 L + g_2}{L + d_2})} - \mu_L(L - b_{LT}(1 - R_F)T) \qquad (8)$$

$$D' = \delta_I I + k_N IF + k_E CI \qquad (9)$$

$$X' = \frac{b_x M \Sigma_2}{\Sigma_2 + (\Sigma_2 + \frac{g_1 L + g_2}{L + d_2})(\frac{k_1 L + k_2}{L + d_1})} - \mu_X X \qquad (10)$$

$$M' = \frac{b_{mk} K^{h_k}}{a_{mk}^{h_k} + K^{h_k}} - \mu_M(M - b_M) \qquad (11)$$

$$K' = \frac{b_K M \Sigma_1}{\Sigma_1 + \frac{(g_1 L + g_2)}{L + d_2}} - \mu_k K \qquad (12)$$

$$[III]' = \frac{\lambda_{III}(X + F)}{h_{III} + (\frac{g_4}{[IIa] + d_{IIa}}) + X + F} - K_S[II][III] - \mu_{III}[III] \quad (13)$$

$$[II]' = -K_S[II][III] - K_P[II][IIa] \qquad (14)$$

$$[IIa]' = K_S[II][III] + K_P[II][IIa] - K_I[IIa][AT] \qquad (15)$$

$$[AT]' = -K_I[IIa][III][AT] \qquad (16)$$

$$Q' = \kappa[IIa][III](1 - Q) \qquad (17)$$

where $\Sigma_1 = a_{11}X + a_{12}D, \Sigma_2 = a_{11}X + a_{12}D + \frac{a_{21}V}{a_{22} + V}$, and $R_F = \frac{F}{a_{RF} + F}$.

TF induction threshold T_{III}: $\lambda_{III} = \begin{cases} 0, & X < T_{III} \\ \lambda_{III0}, & X \geq T_{III} \end{cases}$.

S1.2 Parameter estimation and initial conditions.

Here we include the details of the parameterization of model (1) we develop in the main text. We list model parameters informing the mathematical model (1) in Supplementary Table S3 as well as their respective ranges, units, and sources. Equations (1)-(12) are parametrized using estimates from existing influenza infection modeling studies [21, 22]. Equations (14)-(16) are parametrized using existing studies modelling blood coagulation [12, 23]. We inform the remaining parameters by integrating several additional sources of experimental and clinical data.

Decay of tissue factor μ_{III}: In an *in vitro* experiment, human peripheral blood mononuclear cells (PBMC) were isolated and TF was solubilized from these cells [47]. Using the reported TF half-life of 1.3 hours from this *in vitro* experiment, we estimate the decay of TF μ_{III} to be 12.9 1/day [47].

Induction of TF: We estimate parameters for Equation (13) in model (1). For the generation of tissue factor via TNFα and IFN-I we use *in vitro* experimental data to fit λ_{III} and h_{III} [46]. We also inform the parameters g_4 and d_{IIa}, which relate the synergistic effects of TNFα and thrombin on TF induction, using *in vitro* data [46]. In particular, these parameters were calibrated to reflect a 6.2-fold increase in TF induction due to TNFα when thrombin is present [46]. We note that the data collected from [27, 47] was based on healthy subjects. We did not explicitly consider the health status of individuals in this study; but, it is an important factor that may be critical to explore as discussed in the main text as in Section 3.1 as a future direction.

Baseline maximal uptake of TF λ_{IIIB} *and substrate affinity* h_{III}: We proceed using *in vitro* experimental data measuring TF expression on the surface of epithelial cells [27]. Human umbilical vein cells (HUVECs) were prepared and TF expression was assessed by incubating with TNFα and vascular endothelial growth factor (VEGF) for 6 hours [27]. To inform h_{III} and λ_{III} we use Equation (13) and the following assumptions: *i)* no TF decay over the course of the experiment and *ii)* TNFα is constant over the course of the experiment. Also, IFN-γ is not present in this experiment, so $F = 0$. Hence, we have the following model equation

$$[III]' = \frac{\lambda_{IIIB} X}{(h_{III} + (g_4/d_{IIa}) + X)},$$

with the following simplification

$$[III]' = \frac{\lambda_{IIIB} X}{(h_{III} + X)}.$$

Separating variables results in the following linear equation

$$h_{III} t_f - \lambda_{IIIB} X = -X[III](t_f)$$

with variables λ_{IIIB} and h_{III}. Now, we use the following data points from the outlined experiment data for TF induced by TNFα. We denote the pairs in terms of (TNFα, TF) for two experiments: Experiment 1 (0.015 nM, 700 nM) and experiment 2 (0.2 nM, 1450 nM).

We then solve the corresponding linear system to find $\lambda_{IIIB} = 4.231/min$ and $h_{III} = 0.02$ nM. (Experimental data acquired from [27]). Page 335, Figure 1)

Maximal uptake of TF, λ_{III}: The maximal uptake of TF is enhanced by the presence of thrombin [42]. Here we account for the synergistic effects of thrombin and TNFα on TF induction. We use *in vitro* experimental data which assesses TF expression in HUVECs after incubation with 1) TNFα or 2) both TNFα and thrombin. Hence, we use the baseline value of maximal TF induction λ_{IIIB} to proceed. We increase λ_{IIIB} 6.2-fold to reflect the altered maximal uptake from thrombin presence, hence $\lambda_{III} = 6.2\lambda_{IIIB} = 26.23$ 1/min [46].

Synergistic effects of Thrombin/TNFα: To reflect the observed 6.2-fold reduction in TF induction due to TNFα when thrombin is not present, we set $g4 = 6.2h_{III}$, and $d_{IIa} = 1$ [27].

Synergistic effects of TNFα/IFN-γ: We also capture the synergistic effects of TNFα/IFN-γ on TF induction. IFN-γ has not been shown solely induce TF *in vitro*; however, IFN-γ modulates TF induction due to TNF-α presence [43]. This synergism of TNFα/IFN-γ on TF induction is reflected in the first term of Equation (13) with Michael-Menten form.

Blood clotting severity parameter κ: We inform this parameter which appears in Equation (17) using *in vitro* experimental data [23]. To proceed, we first assume the following: 1) thrombin concentration is constant throughout the course of the experiment and 2) thrombin is spatially homogeneous in the artery. Writing Equation (17) we have

$$Q' = \kappa[IIa][III](1 - Q).$$

Solving this differential equation by separating variables we have

$$ln(\frac{1-Q_0}{1-Q_f}) = [IIa][III]\kappa t_f.$$

Therefore

$$\kappa = ln(\frac{1-Q_0}{1-Q_f})\frac{1}{t_f[IIa][III]}. \qquad \text{(i)}$$

Now, we may use *in vitro* experimental data from a flow chamber and the above relationship to estimate κ [23]. In particular, with a peak thrombin concentration of 800nM and over the course of 1000 seconds 90% vessel blockage had developed. Lastly, we assume that TF remains constant at 0.4 pM to reflect estimates from patients diagnosed with coronary artery disease [48]. Now from Equation (i), we find $\kappa = 0.43$. With 300nM thrombin and over the course of 2700 seconds, a 17% blockage occurs. This case corresponds to κ = 0.035. Hence we estimate κ to be in the interval [0.035, 0.43], giving us a relationship between thrombin, clot size, and TF. Experimental data acquired from Figure 4a in [23].

Initial conditions: The initial conditions for model (1) are given in Supplementary Table S2. For the initial influenza virus dose V and target epithelial cell count we use estimates and experimental data reported in [22]. Blood coagulation initial conditions were chosen to reflect *in vitro* experimental levels [12]. Realistically, circulating prothrombin and antithrombin levels vary person-to-person. High prothrombin levels have been correlated with a risk of arterial and venous thrombosis and are also affected by genetic conditions [20]. We capture these individual effects in our study by incorporating stochasticity in the initial values of prothrombin and thrombin for relevant simulations. In particular, we vary each initial condition by 20% of their baseline value. We begin simulations with model (1) at the disease free equilibrium, with an initial viral load, to reflect an infection of an individual at baseline status. We discuss these details in section 4.2-4.3 of the main text. The initial conditions are shown in Supplementary Table S2.

Threshold level of TNFα to induce TF production: Here we use clinical data to estimate the threshold T_{III}. During a 7-day window within initial influenza infection, an elevated risk of acute myocardial infarction (AMI) has been observed [1]. Among 364 hospitalizations for AMI in 332 patients with laboratory-confirmed influenza, 20 occurred during 7-day post-infection risk window and the remaining 344 occurred outside of the risk window. The weekly admissions per week rates were 3.3 to 20 (incidence ratio 6.05) [1]. To estimate T_{III}, we attribute these AMIs to blood clot formation; hence, given an influenza infection an individual has a probability 20/364 of experiencing an AMI within 7 days. To ensure the clotting frequency given by model (1) is consistent with this clinical data, we use a minimization process to estimate T_{III}.

The process to find optimal T_{III} was conducted as follows. First, let $p_{observed}$ be the observed probability of AMI among study patients in [1] following infection, i.e., $p_{observed} = \frac{20}{364}$. We sample model parameters and initial conditions from their respective ranges (Supplementary Table S3) to generate 364 parameter sets using Latin Hypercube Sampling. Now, let $p(T_{III}) = \frac{n}{364}$, where n is the number of blood clot events in 364 model runs from all parameter sets, for a given threshold value of T_{III}. We then search for an optimal T_{III} such that we minimize the difference between model simulation and clinical observations. This process can be described mathematically as follows.

Let p be the probability of AMI in 7 days. Find T_{III} by solving the following minimization problem:

$$\min_{T_{III}} \quad (p(T_{III}) - p_{observed})$$

We solve this numerically using Matlab's *fminsearch* function. The result is a threshold TNFα level $T_{III} = 27.36$ pg/ml. For more details about Latin Hypercube Sampling and optimization process, see [53].

S1.3 Rationale for IFN-I units

We have adopted the modelling construction of IFN-I kinetics from a prior work [22]. In this work, IFN-I was not directly observed and was left dimensionless. In the present study, we adopt this choice in units.

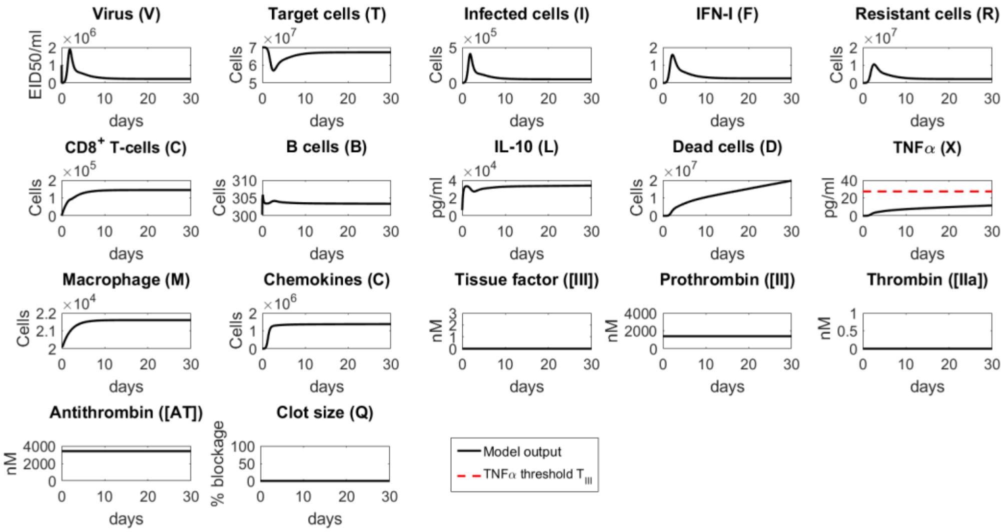

Supplementary Figure S1. Temporal variation of the full model: Influenza infection of an individual at baseline. In this case, the immune response is triggered as a result of acute infection. However, no blood clot is formed as a result as TNFα remains below T_{III}.

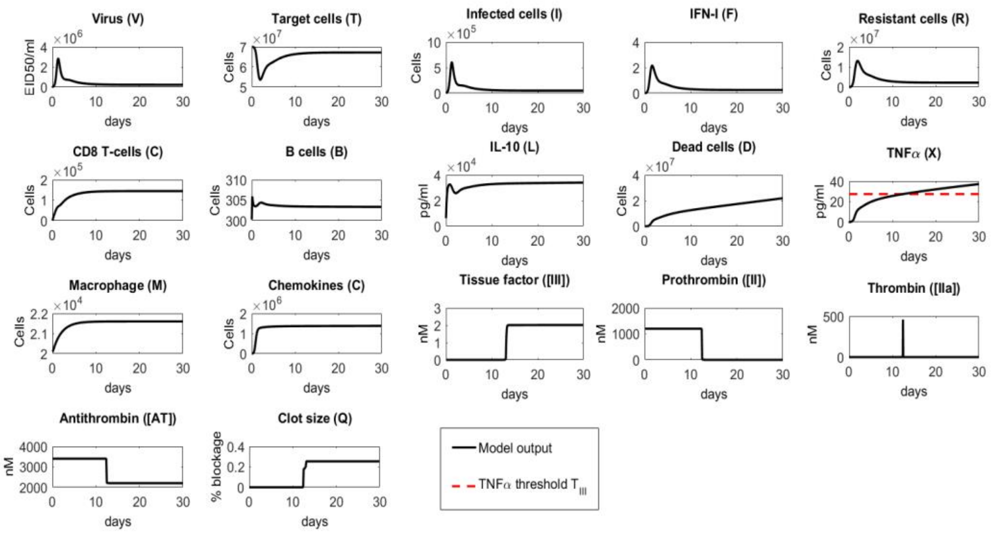

Supplementary Figure S2. Temporal variation of the full model: Influenza infection of an individual experiencing high inflammation (elevated TNFα). In this case, the immune response is triggered as a result of acute infection. Following the immune response TF is induced as the TNFα level crossed the threshold. A minor blood clot is formed at day 15 post-infection, resulting in roughly 0.2% blockage of an artery. We outline simulation procedure and numerical solution in section 4.3 in the main text**.**

Supplementary Table S1: Switching conditions for parameters involved in blood coagulation. Rates for phases propagation, initiation, and inhibition depend on thrombin [IIa] levels relative to 2 nM. For details of the simplified extrinsic pathway and corresponding model formulation for blood coagulation see [12].

	$[IIa] < 2$ nM	$[IIa] \geq 2$ nM
K_S	k_s	0
K_I	k_{i2}	k_{i1}
K_P	0	k_p

Supplementary Table S2. Model variables: Brief description, units, and initial conditions for state variables of model (1).

Model variable	Description	Units	Initial condition
V	Virus	EID $_{50}$/ml	10^4 [22]
T	Target epithelial cell	Cells	7×10^7 [22]
I	Infected epithelial cell	Cells	0
F	Interferon-I	dimensionless	0
R	Resistant epithelial cell	Cells	0
C	$CD8^+$ T cell	Cells	0
B	B cell	Cells	$\frac{(b_P+b_{BP}b_M b_{LT}Tb_{max}/\mu_L)}{(b_{BP}b_M+b_{LT}T/\mu_L+\mu_B)}$
L	Interleukin-10	pg/ml	$b_{LT}\mathrm{T}_0/\mu_L$
D	Dead cell	Cells	0
X	TNFα	pg/ml	0
M	Macrophage	cells	b_M
K	Chemokines	cells	0
$[III]$	Tissue Factor	nM	0
$[IIa]$	Thrombin	nM	0
$[II]$	Prothrombin	nM	[1120,1680] (baseline 1400 [23])
$[AT]$	Antithrombin	nM	[2720,4080] (baseline 3400 [23])
Q	Clot size	%	0

Supplementary Table S3. Model parameter ranges: a brief description, values and units, and reference associated with each parameter for model (1).

Parameter	Description	Value and units	Baseline value (if different than range)	Reference
V_0	initial viral load	10^4 EID $_{50}$/ml		[22]
T_0	Initial number of epithelial cells in the URT	7×10^7 cells		[22]
g_T	Base growth rate of healthy cells	0.8 d $^{-1}$		[22]
p_V	Viral production rate	210 $[u_V]$ cell $^{-1}$d $^{-1}$		[22]
p_F	IFN production rate	10^{-5} $[u_F]$cell $^{-1}$d $^{-1}$		[22]
δ_V	Non-specific viral clearance rate	5d $^{-1}$		[22]
δ_I	Non-specific death rate of infected cells	2 d $^{-1}$		[22]
δ_F	IFN degradation rate	2 d $^{-1}$		[22]
β	Rate of viral consumption by binding to target cells	5×10^{-7}cell $^{-1}d^{-1}$		[22]
β'	Rate of infection of target cells by virus	3×10^{-8} $[u_V]^{-1}$ d $^{-1}$		[22]
ϕ	Rate of conversion to virus-resistant state	0.33 $[u_F]^{-1}$ d $^{-1}$		[22]
ρ	Rate of recovery from virus-resistant state	2.6 d $^{-1}$		[22]
κ_N	Killing rate of infected cells by IFN-activated NK cells	$2.5[u_F]^{-1}d^{-1}$		[22]
κ_E	Killing rate of infected cells by effector CD8 $^+$ T cells	5×10^{-5}cells $^{-1}d^{-1}$		[22]
b_P	Baseline number of activated APC	$[3.0 \times 10^2, 9.6 \times 10^3]$ cells	3.0×10^2	[21]
b_{BP}	APC induced activation of B cells	$[6.0 \times 10^{-8}, 2.4 \times 10^{-5}]$ ml/(cell pg d)	3.03×10^{-6}	[21]
b_{max}	Maximum number of B cells	3.0×10^2 cells		[21]
μ_B	Decay/removal of B cells	$[5.0 \times 10^{-2}, 8.0 \times 10^{-1}]$ d $^{-1}$	6.45×10^{-1}	[21]
b_L	Maximal production rate of IL-10 by macrophages	$[2.1 \times 10^{-2}, 8.3]$ pg/ml/d	2.14	[21]
M	Baseline number of macrophages	$[3.0 \times 10^2, 1.2 \times 10^4]$ cells	3.0×10^2	[21]
g_1	Inhibitory term for effect of IL-10 on cytokine production	$[1.1, 4.5 \times 10^2]$	2.89×10^2	[21]
g_2	Inhibitory term for effect of IL-10 on cytokine production	$[3.4 \times 10^2, 1.3 \times 10^5]$	2.96×10^3	[21]
d_1	Inhibitory term for effect of IL-10 on cytokine production	$[2.5 \times 10^1, 4.0 \times 10^2]$	4.54×10^1	[21]
d_2	Inhibitory term for effect of IL-10 on cytokine production	$[1.89 \times 10^2, 4.68 \times 10^2]$	4.13×10^2	[21]
μ_L	Decay/removal of IL-10	$[1.8, 1.2 \times 10^1]$ d $^{-1}$	5.23	[21]
b_{LT}	Production rate of IL-10 by target epithelial cells	$[5.0 \times 10^{-5}, 1.0 \times 10^{-3}]$ pg/ml/Cell	4.78×10^{-4}	[21]
k_1	Inhibitory term for pro-inflammatory cytokine production	$[2.5 \times 10^{-1}, 2.0 \times 10^1]$	1.59	[21]
k_2	Inhibitory term for pro-inflammatory cytokine production	$[2.5, 4.0 \times 10^2]$ pg/ml	1.18×10^2	[21]
μ_X	Decay/removal of TNFα	$[2.5 \times 10^1, 7.2 \times 10^2]$ d^{-1}	5.28×10^2	[21]
a_{11}	Signal induced to macrophages by TNFα	5.5×10^{-5} ml/pg		[21]
a_{12}	Signal induced to macrophages by damaged epithelial cells	4.0×10^{-6} Cells $^{-1}$		[21]

a_{21}	Maximal signal induced to macrophages by virus	$[1.6, 6.4 \times 10^2]$	1.6	[21]
a_{22}	Substrate affinity for signal induced by virus	6.0×10^5 pfu/ml		[21]
a_{RF}	Substrate affinity of epithelial cells to IFN-α/β	$[1.0 \times 10^1, 1.4 \times 10^2]$ pg/ml	8.07×10^1	[21]
h_c	Hill coefficient for T cell production		2	[21]
a_{cp}	Substrate affinity in the activation of effector cells	$[1.5 \times 10^3, 6.0 \times 10^4]$ Cells	2.56×10^3	[21]
b_{ei}	Removal of effector cells during infected cell elimination	3.0×10^{-5} Cell $^{-1}$ d $^{-1}$		[21]
μ_C	Decay/removal of effector cells	$[1.0 \times 10^{-1}, 7.0 \times 10^{-1}]$ d $^{-1}$	5.56×10^{-1}	[21]
b_{cp}	Maximal activation rate of effector cells	$[1.0 \times 10^4, 4.0 \times 10^5]$ Cells d $^{-1}$	8.23×10^4	[21]
b_{fp}	Production rate of IFN-α/β by APC	$[4.7 \times 10^{-4}, 1.9 \times 10^{-1}]$	1.56×10^{-1}	[21]
b_{mk}	Maximal chemotactic adduction of macrophages	$[7.5 \times 10^2, 4.1 \times 10^4]$	7.5×10^2,	[21]
h_k	Hill coefficient for the recruitment of macrophages	3		[21]
μ_M	Death rate of macrophages	$[1.0 \times 10^{-2}, 5.0 \times 10^{-1}]$	4.99×10^{-1}	[21]
b_M	Baseline number of macrophages	$[3.0 \times 10^2, 1.2 \times 10^4]$ cells	3.0×10^2	[21]
b_K	Maximal production rate of chemokines	4.0pg/ml/d		[21]
μ_K	Decay/removal of chemokines	$[1.5 \times 10^1, 1.8 \times 10^2]$ d $^{-1}$	1.16×10^1	[21]
g_{va}	Elimination of virus due to antibody neutralization	3.0×10^{-2} ml/pfu/d		[21]
b_x	Maximal production rate of TNF-α	$[7.8 \times 10^{-2}, 3.1 \times 10^1]$	1	[21]
a_{mc}	Substrate affinity for adduction of macrophages	$[1.3 \times 10^2, 2.0 \times 10^3]$	5.0×10^{-2}	[21]
k_s	Rate constant for thrombin initiation	5×10^{-3} nM $^{-1}$s $^{-1}$		[12]
k_p	Rate constant for thrombin propagation	4.155×10^{-5} nM $^{-1}$s $^{-1}$		[12]
k_{i1}	Rate constant for thrombin inhibition	6.347×10^{-6} nM $^{-1}$s $^{-1}$		[12]
k_{i2}	Rate constant for thrombin inhibition	1.9551×10^{-7} nM $^{-1}$s $^{-1}$		[12]
λ_{III}	Recruitment of TF due to TNFα	26.23 nM d $^{-1}$		Estimated [46]
μ_{III}	Death rate of TF	12.9 d $^{-1}$		Estimated [47]
h_{III}	Substrate affinity in the production of TF	0.02 nM		Estimated [27]
g_4	Inhibitory term for effect of thrombin on TF production	0.124		Estimated [46]
d_{IIa}	Inhibitory term for effect of thrombin on TF production	1		Estimated [46]
κ	Growth rate of clot proportion	$[0.035, 0.43]$ 1/(day nM ^{2})	0.43	Estimated [23]
T_{III}	Threshold level of TNFα for TF induction	27.36 pg/nM		Estimated [1]
